# Differential Clearance of Aβ Species from the Brain by Brain Lymphatic Endothelial Cells in Zebrafish

**DOI:** 10.3390/ijms222111883

**Published:** 2021-11-02

**Authors:** Yun-Mi Jeong, Jae-Geun Lee, Hyun-Ju Cho, Wang Sik Lee, Jinyoung Jeong, Jeong-Soo Lee

**Affiliations:** 1 Disease Target Structure Research Center, Korea Research Institute of Bioscience and Biotechnology, 125 Gwahak–ro, Yuseong-gu, Daejeon 34141, Korea; angdoym@kribb.re.kr (Y.-M.J.); jglee89@kribb.re.kr (J.-G.L.); alleles@kribb.re.kr (H.-J.C.); 2 Dementia DTC R&D Convergence Program, Korea Institute of Science and Technology, Hwarang-ro 14-gil 5, Seongbuk-gu, Seoul 02792, Korea; 3 Department of Functional Genomics, KRIBB School, University of Science and Technology, 217 Gajeong-ro, Yuseong-gu, Daejeon 34113, Korea; 4 Environmental Disease Research Center, Korea Research Institute of Bioscience and Biotechnology, 125 Gwahak-ro, Yuseong-gu, Daejeon 34141, Korea; wang3026@kribb.re.kr (W.S.L.); jyjeong@kribb.re.kr (J.J.); 5 Department of Biotechnology, KRIBB School, University of Science and Technology, 217 Gajeong-ro, Yuseong-gu, Daejeon 34113, Korea

**Keywords:** Alzheimer’s disease, amyloid beta, brain lymphatic endothelial cells, zebrafish model

## Abstract

The failure of amyloid beta (Aβ) clearance is a major cause of Alzheimer’s disease, and the brain lymphatic systems play a crucial role in clearing toxic proteins. Recently, brain lymphatic endothelial cells (BLECs), a non-lumenized lymphatic cell in the vertebrate brain, was identified, but Aβ clearance via this novel cell is not fully understood. We established an in vivo zebrafish model using fluorescently labeled Aβ42 to investigate the role of BLECs in Aβ clearance. We discovered the efficient clearance of monomeric Aβ42 (mAβ42) compared to oligomeric Aβ42 (oAβ42), which was illustrated by the selective uptake of mAβ42 by BLECs and peripheral transport. The genetic depletion, pharmacological inhibition via the blocking of the mannose receptor, or the laser ablation of BLECs resulted in the defective clearance of mAβ42. The treatment with an Aβ disaggregating agent facilitated the internalization of oAβ42 into BLECs and improved the peripheral transport. Our findings reveal a new role of BLECs in the differential clearance of mAβ42 from the brain and provide a novel therapeutic strategy based on promoting Aβ clearance.

## 1. Introduction

Alzheimer’s disease (AD), the most prevalent form of dementia, is a devastating neurodegenerative disease characterized by cognitive function impairment and memory loss. The pathological characteristics of AD include the extracellular deposition of amyloid beta (Aβ) and neurofibrillary tangles from tau phosphorylation [1]. Although the steady level of Aβ is balanced between production and clearance with the dynamic aggregation and disassembly of Aβ under normal conditions, an imbalance of the Aβ level via perturbed clearance is considered to be a major initiating factor of late onset AD [2,3]. Such an imbalance results in the accumulation of monomeric Aβ (mAβ) peptides in the brain, which then generate oligomers, fibrils, and plaques of Aβ. Oligomeric Aβ (oAβ) is thought to be the most toxic form of Aβ species responsible for AD [4]. These accumulated Aβ species are fundamental as biomarkers for an in vivo diagnosis of AD by measuring their levels in the cerebrospinal fluid (CSF) or performing an amyloid-positron emission tomography (PET) [5]. Thus, it is of great importance to elucidate how different forms of Aβ are cleared under normal and pathological conditions to understand the pathogenesis of AD and to develop an effective therapeutic strategy for AD by reducing the excessive deposition of toxic Aβ species.

The clearance of Aβ from the brain occurs via intricate clearance systems consisting of two major pathways: the blood circulatory clearance, which transports Aβ into the periphery, and the degradation of Aβ by proteases, such as neprilysin, or intracellular degradation by glial cells in the brain [6]. The blood circulatory clearance pathway includes the transport of wastes from the brain parenchyma across the blood–brain barrier (BBB), the bulk flow of interstitial fluid (ISF), and the absorption of CSF into the blood circulatory or lymphatic system [7]. The Aβ cleared from the brain into the periphery are eventually degraded by the red blood cells and macrophages in the circulation or by peripheral organs, such as the liver and kidney [6]. Although earlier mouse studies suggested that the majority of extracellular Aβ was cleared via the transportation across the BBB, recent functional studies on the glymphatic (glial + lymphatic) system in the perivascular network and meningeal lymphatic vessels in the dura mater of the brain showed more complicated clearance routes [7,8,9,10,11]. In particular, recent functional studies revealed that the meningeal lymphatic vessels played critical roles in the drainage of CSF wastes, including Aβ, suggesting a dysfunction of the meningeal lymphatic vessels resulting in a defective Aβ clearance in the brain, which is a key aggravating factor in AD pathology [12,13].

Unlike meningeal lymphatic vessels in the dura mater (the most outer layer of the meninges), the lymphatic vessels of leptomeninges, including the arachnoid and pia mater, were not studied. However, studies in zebrafish identified a distinct non-lumenized meningeal lymphatic cell population, called brain/mural lymphatic endothelial cells (BLECs/muLECs), fluorescent granule perithelial cells (FGPs), or Mato cells that express multiple lymphatic markers [14,15,16] (hereafter denoted as BLECs). Recently, these BLECs were also found in mouse and human leptomeninges, sharing morphological features and molecular markers [17]. BLECs are able to internalize macromolecules such as Aβ40 from the brain or take up lipids with a close proximity to the vasculature, suggesting their involvement in the brain clearance system [14,16,17,18]. However, the exact role of BLECs in AD pathology and the mechanism by which BLECs clear different forms of Aβ species remain to be determined.

In the current study, we established an in vivo zebrafish larval model to investigate the function of BLECs in the clearance of Aβ42, by taking advantage of the transparency of the zebrafish larval brain using cell type-specific fluorescent live reporter lines. Using this model, we performed in vivo, real-time analyses of the initial accumulation followed by the clearance of fluorescently labeled Aβ42 depending on their aggregation status; that is, the mAβ42 or oAβ42 forms. We found that mAβ42 was readily cleared from the brain by the absorption into BLECs, eventually accumulating in the peripheral pronephros for excretion, whereas oAβ42 remained mostly unchanged inside the brain. The BLEC depletion by *ccbe1* (collagen and calcium binding EGF domains 1) gene knockdown or selective cell ablation using a laser, or the pharmacological inhibition of BLEC function using a mannose receptor agonist, exhibited a reduced pronephric accumulation of mAβ42. These results support a specific role of BLECs in the clearance of mAβ. The treatment with an Aβ disaggregating small-molecule EPPS [4-(2-hydroxyethyl)-1-piperazinepropanesulfonic acid] enhanced the BLEC localization and transport of disaggregated oAβ42 into the peripheral pronephros, corroborating the selectivity of mAβ42 clearance by BLECs. Taken together, our analyses reveal a role of recently identified lymphatic cells and BLECs in selectively clearing mAβ42 from the brain in vivo. This finding furthers our understanding of the clearance mechanisms of Aβ in vivo and provides an in vivo platform and a strategy to discover and validate novel AD therapies based on modulating the functionality of BLECs for efficient Aβ clearance.

## 2. Results

### 2.1. Monomeric and Oligomeric Aβ42 Are Differentially Cleared from the Brain

**Figure 1:**
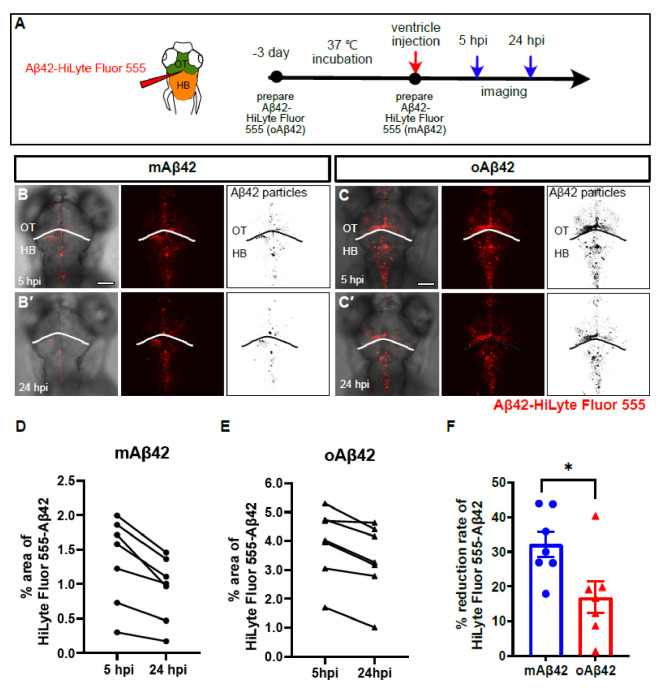
Monomeric Aβ42 peptides are more efficiently cleared from the brain than oligomeric Aβ42. (**A**) A schematic diagram of experimental setup. Fluorescently labeled Aβ42 prepared at different time points were injected into the brain followed by in vivo imaging at 5 hpi and 24 hpi. (**B**,**C′**) Distribution of injected, fluorescently labeled Aβ42 (mAβ42 or oAβ42) in the brains of 3 dpf larvae at 5 hpi (**B**,**C**) and 24 hpi (**B′**,**C′**) (left, overlays with brightfield; middle, HiLyte Fluor 555; right, thresholded images of Aβ42 fluorescence). (**D**,**E**) Quantification of the area fraction (%) occupied by Aβ42 fluorescence within square unit (512 × 512 pixels) at different time points. (**F**) Clearance rate of mAβ42 and oAβ42 between 5 hpi and 24 hpi by fluorescence quantification. Two-tailed unpaired *t*-test, *p* = 0.023. Data are presented as mean ± SEM. N = 7 per group. Data are representative of at least three independent experiments. dpf, days post fertilization; hpi, hours post injection; HB, hindbrain; OT, optic tectum, Scale bars = 100 μm. * *p* < 0.05.

To investigate the role of the BLECs in Aβ42 clearance in vivo, we first established a zebrafish larval model for monitoring Aβ clearance by the cerebroventricular injection of a fluorescently labeled Aβ42 into the zebrafish larval brain. We prepared two kinds of Aβ peptides, mAβ42 and oAβ42, to compare the clearance efficiency using our model. The oligmeric form of the Aβ peptide is thought to be the most toxic species and resistant to clearance [4,19]. The fluorescently labeled oAβ42 was prepared by incubating the fluorescently labeled mAβ42 at 37 °C, according to the previously reported protocol [20]. Fluorescently labeled Aβ42 was shown to exhibit structures and is functionality comparable to unlabeled Aβ42 under an aggregation condition [21]. We confirmed the respective structures by using atomic force microscopy (AFM) (Figure S1). The mAβ42 or oAβ42 peptides were introduced by a microinjection into the brain ventricle between the optic tectum and the hindbrain of zebrafish larvae at 3 days post-fertilization (dpf), and the clearance of the peptides from the brain was followed at 5 and 24 h post-injection (hpi) for a quantification based on the fluorescence area (Figure 1A). The fluorescence of injected mAβ42 or oAβ42 was observed along the brain structures, such as the optic tectum and the hindbrain region (Figure 1B–C′). The mAβ42 fluorescence decreased by more than 30% from 5 hpi to 24 hpi (Figure 1B,B′,D,F). In contrast, the clearance rate of oAβ42 was significantly reduced compared to that of mAβ42 (32.12 ± 3.6% mAβ42 injection vs. 16.90 ± 4.6% oAβ42 injection, *p* = 0.0233, two-tailed unpaired *t*-test, *n* = 7 per group) (Figure 1C,C′,E,F). Such differential clearance efficiency of Aβ42, depending on the aggregation status (a rapid or slowed clearance of mAβ42 or oAβ42, respectively), was similar to the previous observation of the clearance of mAβ40 or oAβ40 in the mouse brain [22].

To evaluate the cellular toxicity induced by different forms of Aβ42 in the brain of zebrafish larvae, we analyzed the cell death after an Aβ42 injection by acridine orange staining, which labeled the condensed chromatins [23]. The injection of oAβ42 induced massive cell death in the brain, especially in the hindbrain, whereas the injection of mAβ42 did not (Figure S2). The fluorescence by fluor-labeled Aβ42 was co-localized with the anti-pan-β-amyloid antibody (4G8), confirming that the fluorescence represented the actual Aβ42 peptides (Figure S3A–D). Taken together, these results reveal the differential clearance of Aβ, depending on its aggregation status, using a successfully established acute zebrafish larval model that can visualize and monitor the clearance dynamics of Aβ42 in the brain in vivo and in real time. This acute Aβ42 injection zebrafish model can be utilized as a unique, experimental in vivo platform to investigate the brain clearance system regarding Aβ42 removal in real time and under in vivo conditions.

### 2.2. Cleared mAβ42 Accumulates in the Pronephros via Blood Flow

**Figure 2:**
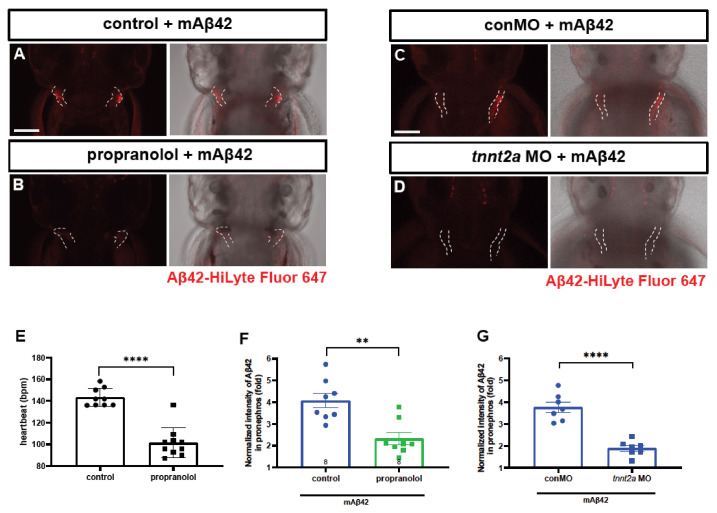

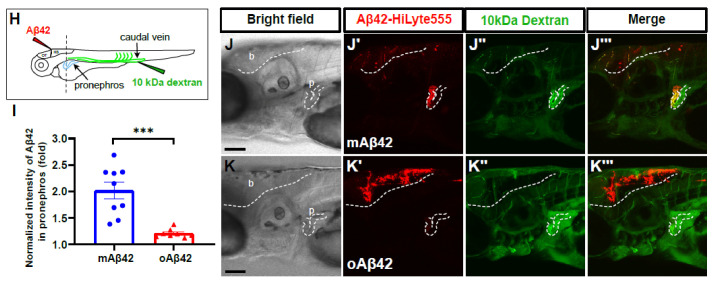
Cleared mAβ42 accumulates in the pronephros via blood flow. (**A**,**B**) mAβ42-injected larvae after propranolol (100 μM) treatment. The mAβ42 intensity in the pronephros of propranolol-treated larvae (white dotted lines) decreased (**B**) compared to control (**A**). (**C**,**D**) mAβ42-injected larvae after control morphants (**C**) and 200 μM *tnnt2a* morphants (**D**). (**E**) Quantification of heartbeats upon propranolol treatment. *n* = 9 for control group, *n* = 10 for propranolol. (**F**) Quantification of the mAβ42 intensity in the pronephros after propranolol treatment. *n* = 8 per group. (**G**) Quantification of the mAβ42 intensity in the pronephros of control and *tnnt2a* morphants. Statistical significance was determined by two tailed unpaired *t*-test. Data are presented as mean ± SEM. *n* = 9 per group. Data are representative of at least three independent experiments. (**H**) A schematic diagram of experimental setting of Aβ and tracer injection. mAβ42 or oAβ42 (HiLyte) was injected into ventricle and 10 kDa Dextran was injected into caudal vein. (**I**) Quantification of the Aβ42 fluorescence intensity in the pronephros normalized by background fluorescence. *n* = 9 for mAβ42, *n* = 8 for oAβ42. Data are representative of at least three independent experiments. (**J**–**K‴**) Confocal fluorescence images showing the brain and pronephros of zebrafish with lateral view after mAβ42 (**J**) or oAβ42 (**K**) injection at 3 dpf. Red fluorescence indicates Aβ42-HiLyte Fluor and green fluorescence show 10 kDa dextran tracer injected into caudal vein. (**J′**) Ventricle-injected mAβ42 was seen in the pronephros region (white dotted lines and depicted as p). (**K′**) Ventricle-injected oAβ42 was seen only in the brain region (b), but not detected in the pronephros. Caudal vein-injected dextran accumulated in the pronephros of both mAβ42 and oAβ42-injected larvae (**J″**,**K″**). (**J‴**,**K‴**) show merged images. b, brain; MO, morpholino; p, pronephros; Scale bars = 100 μm. ** *p* < 0.001; *** *p* < 0.0005; **** *p* < 0.0001.

We next investigated whether the cleared fluorescent Aβ42 from the brain was transported into the periphery in our Aβ clearance model. The kidney is considered to be one of the major peripheral organs for Aβ clearance in both human and animal studies [6,24]. Recently, Tian and colleagues showed that the kidney removed Aβ from the blood, and proposed the pathophysiological significance of the renal clearance of Aβ in mammals [25]. We observed a similar clearance as a consequence of Aβ in the zebrafish pronephros (Figure 2), a functional kidney counterpart in the larval stages consisting of the glomerulus and bilateral pronephric tubules fused with the vascular structure at the midline [26]. We observed the accumulation of mAβ42 in the pronephric tubules at 4 hpi, suggesting that mAβ42 introduced via injection was promptly cleared from the brain and transported into the pronephros, as a main periphery organ for clearance in our model (Figure 2A,J′ and Figure S4C,D). In contrast, the fluorescence of oAβ42 in the pronephros was significantly reduced (Figure 2K′ and Figure S4E,F), with a reduction greater than 80% (two-tailed unpaired *t* test, *p* = 0.0002), compared to that of mAβ42 (Figure 2I). The accumulation of mAβ42 and oAβ42 in the pronephros shown by the fluorescence was also confirmed by an anti-pan-β-amyloid antibody (4G8) analysis (Figure S3E,F).

To verify that the pronephric accumulation of mAβ42 was mediated by blood flow, we dampened the heartbeat by using propranolol treatment and checked whether the pronephric accumulation of mAβ42 was affected (Figure 2A,B). The treatment with propranolol, a non-selective β-adrenergic receptor blocker, resulted in a significant decrease in the heart rate (Figure 2E). The injection of mAβ42 into the propranolol-treated larval brain resulted in a significant decrease (~43% reduction; two-tailed unpaired *t* test, *p* = 0.0013; *n* = 8 per group) in the pronephric accumulation of mAβ42 compared to the control (Figure 2A,B,F), suggesting that the pronephric accumulation of mAβ42 in our model depended on blood flow. To rule out the potential unintended side effects of propranolol, we alternatively blocked the heartbeat using the morpholino against *tnnt2a*, an essential gene for heartbeat [27]. Upon *tnnt2a* knockdown, the pronephric accumulation of brain-injected mAβ42 was also significantly decreased (~49% reduction, two-tailed unpaired *t* test, *p* < 0.0001; *n* = 9 per group) compared to the control (Figure 2C,D,G).

To further confirm that the introduced mAβ42 in the brain may be transported to the pronephros via blood circulation in our model, we first injected mAβ42 or oAβ42 into the ventricle of the brain at 3 dpf, followed by the injection of fluorescently labeled 10 kDa dextran as a tracer molecule into the caudal vein of the same larvae, which could be visualized throughout the vasculature and taken up by the pronephric tubules [28] (Figure 2H). We observed that both the brain ventricle-injected mAβ42 and the peripheral vein-injected tracer were co-localized in the pronephric tubule (Figure 2J–J‴, red and green colors, respectively). In contrast, oAβ42 injected larvae showed a low and weak accumulation even when the tracer was highly visible in the pronephros (Figure 2K–K‴). The transverse sections at the level of the pronephric tubules of these larvae also showed an uptake of 10 kDa-dextran together with mAβ42, but not with oAβ42 (Figure S4H,I). Taken together, these results implied a blood circulatory clearance system through which mAβ42, but not oAβ42, was transported from the brain into the peripheral tissue for degradation and excretion.

### 2.3. Monomeric Aβ42 Is Taken up by Brain Lymphatic Endothelial Cells

**Figure 3:**
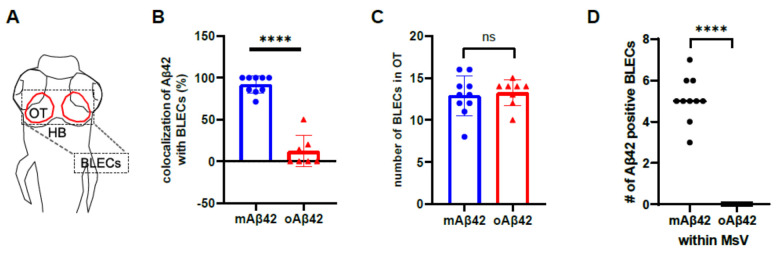

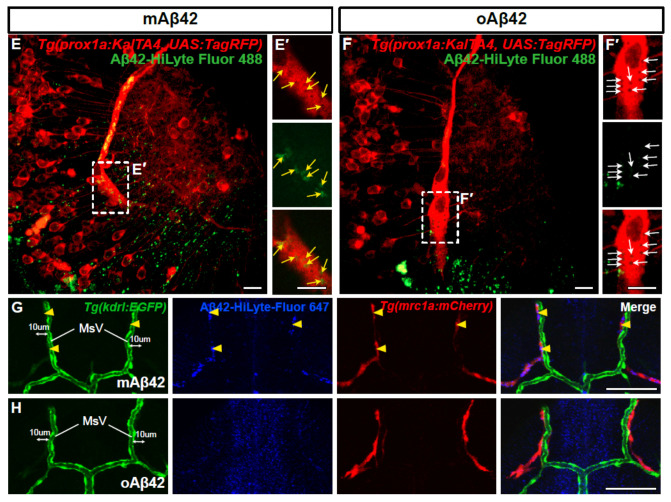
Brain lymphatic endothelial cells take up monomeric Aβ42, but not oligomeric Aβ42. (**A**) A schematic diagram of the larval brain with the dorsal view. Red lines in the dotted box depict the loop structure of the brain lymphatic endothelial cells (BLECs) in the optic tectum. (**B**) Quantification of the co-localization of Aβ42 with BLECs. Data are presented as mean ± SEM. *n* = 9 for mAβ42 and *n* = 7 for oAβ42. Statistical significance was determined by two-tailed unpaired *t*-test. (**C**) Quantification of the numbers of *mrc1a*+ BLECs in the optic tectum after mAβ42 or oAβ42 injection in the *Tg(mrc1a:mCherry)*. Data are presented as mean ± SEM. *n* = 9 per group. (**D**) Quantification of the number of Aβ42 positive BLECs nearby (within 10 μm) mesencephalic vein (MsV) region showing *mrc1a+* positivity. Statistical significance was determined by ordinary one-way ANOVA with Tukey’s test. *n* = 10 for mAβ42 and *n* = 8 for oAβ42. (**E**,**F**) Confocal projections of *prox1a:RFP+* BLECs and Aβ42-HiLyte Fluor 488. (**E′**,**F′**) High magnification of dotted boxes in (**E**,**F**) showing endocytic vesicles of BLECs. mAβ42-injected BLECs showed robust uptake of mAβ42 into endocytic vesicles ((**E′**), yellow arrows) whereas oAβ42-injected did weak uptake ((**F′**), white arrows). Scale bars in (**E**–**F′**) = 10 μm. (**G**,**H**) Confocal fluorescence images of the brain optic tectum region with double transgenic *Tg*(*kdrl:EGFP*); *Tg*(*mrc1a:mCherry*) larvae after Aβ42 injection (HiLyte Fluor 647-Aβ42) at 3 dpf. mAβ42 fluorescence detected in the neighboring (within 10 μm) *kdrl:EGFP*+ cerebrovasculature was mostly co-localized with *mrc1a:mCherry+* BLECs (yellow arrowheads) (**G**), whereas oAβ42 (**H**) fluorescence neighboring *kdrl:EGFP*+ cerebro-vasculatures was not. HB, hindbrain; OT, optic tectum; MsV, mesencephalic vein; Scale bars in (**G**,**H**) = 50 μm. ns, not significant; **** *p* < 0.0001.

Recently identified brain lymphatic endothelial cells (BLECs) may be implicated in the clearance of brain waste due to their anatomical proximity to the cerebrovasculature and their capability to internalize macromolecules from the brain [16]. However, the role of BLECs in amyloid pathology and the clearance of toxic Aβ is not fully defined. In order to investigate the role of BLECs in Aβ clearance, we performed the cerebroventricular injection of mAβ42 or oAβ42 into transgenic lines, *Tg*(*prox1a:KalTA4, UAS:TagRFP*) and *Tg*(*mrc1a:mCherry*), which expressed red fluorescent proteins in the lymphatic system that visualized 5~10 BLECs in the loop structure of the optic tectum at 3 dpf [29,30] (Figure 3A,E–H). Upon mAβ42 injection, the Aβ42 fluorescence (HiLyte Fluor 488 or 647) in the optic tectum was distinctly co-localized with *prox1a-* or *mrc1a*-positive cells in the BLEC loop (Figure 3B,E: the co-localization frequency of mAβ42 in *prox1a*+ BLECs = 91.80%, *n* = 9). In particular, the robust uptake of mAβ42 into the endocytic vesicles in BLECs was observed with high-resolution confocal imaging (Figure 3E, yellow arrows in Figure 3E′). In contrast, the co-localization of oAβ42 in BLECs was quite scarce following oAβ42 injection (Figure 3B,F: the co-localization frequency of oAβ42 in *prox1a*+ BLECs, 12.04%), and the endocytic vesicles in BLECs were barely positive with oAβ42 fluorescence (Figure 3F, white arrows in Figure 3F′). To validate that the injected mAβ42 was internalized into endocytic vesicles, we used pHrodo Green dextran (pHrodoGreen), which emitted a pH-sensitive fluorescence upon its internalization in the acidic environment and was used to reveal the intracellular uptake in BLECs [16,18]. After mAβ42 or oAβ42 injection, following pHrodo injection (Figure S5A), we observed the co-localization of mAβ42 labeled with HiLyte Fluor 647 (mAβ42-HiLyte Fluor 647) and pHrodo in BLECs (Figure S5B), while oAβ42 was undetected in BLECs (Figure S5C), indicating that the mAβ42 was actively internalized into the endocytic compartment of BLECs.

To further confirm that this differential Aβ internalization, according to its aggregation status, occurs in BLECs but not in blood vessels in the brain, we examined the co-localization of fluorescent Aβ using the double transgenic line *Tg*(*mrc1a:mCherry*); *Tg*(*kdrl:EGFP*) to visualize BLECs and endothelial cells simultaneously. The majority of mAβ42-HiLyte Fluor 647 that co-localized with *mrc1a*-positive BLECs in the optic tectum were neighbored by, but not overlapped with, *kdrl*-positive endothelial cells (magenta with yellow arrowheads in Figure 3G and Figure S6C). In contrast, oAβ42 was not overlapped with either *mrc1a:mCherry*-positive BLECs or the *kdrl:EGFP*-positive endothelial cells (Figure 3F,H and Figure S6B,D). Similar to the optic tectum region, mAβ42 in the hindbrain was also detectable adjacent to the primordial hindbrain channels of *Tg*(*kdrl:EGFP*) (arrows in Figure S6E), whereas oAβ42 was seen only in the brain region devoid of the vasculature (Figure S6B,F). To determine whether the mAβ42 was located in BLECs nearby vasculatures, we quantified the number of Aβ-positive BLECs colocalized with *mrc1a*-positive BLECs within 10 μm of the paired mesencephalic vein (MsV), located in the dorsal midline of the brain (Figure 3D,G,H). The majority of mAβ42 fluorescence nearby the MsV coincided with *mrc1a*-positive cells, whereas oAβ42 hardly overlapped with them (Figure 3D: ordinary one-way ANOVA with Tukey’s test, *p* < 0.0001; *n* = 10 for mAβ42 and *n* = 8 for oAβ42). These suggest that the vasculature-associated BLECs participate in the selective internalization of mAβ42 but not of oAβ42.

Since Aβ oligomers are known to have toxicity with various mechanisms [31], the failure of oAβ42 internalization into BLECs may have occurred due to the toxic effect of oAβ42 damaging BLECs. To exclude such a possibility, we counted the number of BLECs in the optic tectum of the *Tg*(*mrc1a:mCherry*) after mAβ42 or oAβ42 injection. The number, as well as the gross shape, of BLECs in the optic tectum was comparable in both conditions (Figure 3C: compare Figure 3E,F), suggesting that the oAβ42 did not impair the anatomy and survival of BLECs. Thus, the decline in the internalization of oAβ42 into BLECs is likely due to the properties of oAβ42, such as the size or structures of oligomers preventing its internalization into BLECs and their removal by the brain clearance system once Aβs are aggregated, which may explain Aβ oligomer-specific accumulation and toxicity in AD pathogenesis.

The clearance of mAβ42 in the BLECs from the brain was further confirmed by observing the fade-out of mAβ42 localized in *mrc1a:mCherry*-positive BLECs using the double transgenic line *Tg*(*mrc1a:mCherry*); *Tg*(*kdrl:EGFP*) in real time (Supplementary Video S1, Figure S7). Taken together, these data indicate that BLECs in the brain have a preference to internalize mAβ42 over oAβ42 and participate in clearing the mAβ42 from the brain.

### 2.4. BLEC Depletion Reduces the Peripheral Transport of mAβ42 to the Pronephros

**Figure 4:**
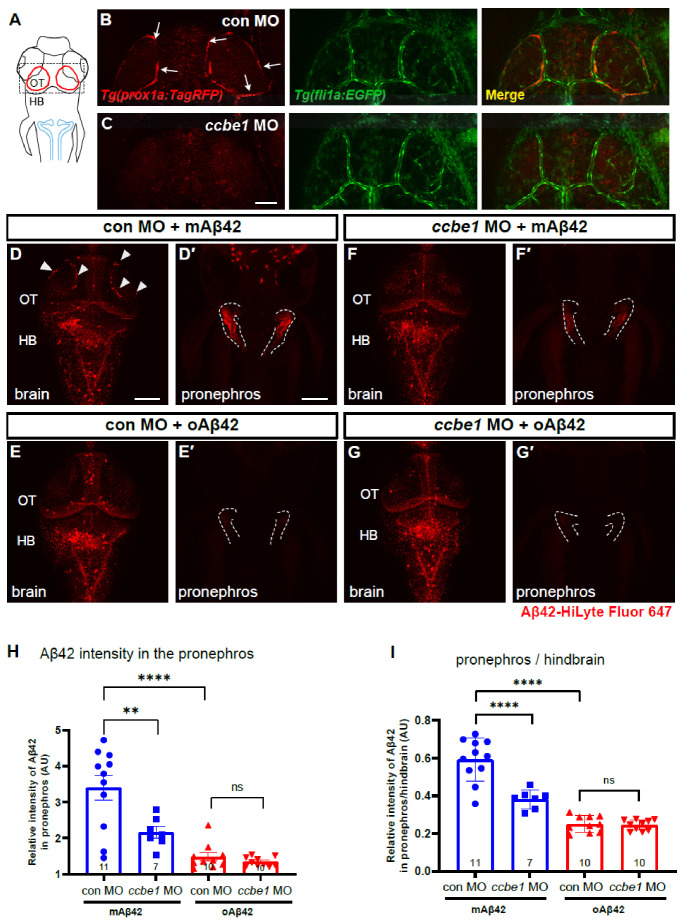
BLECs depletion decreases peripheral transport of mAβ42 to the pronephros. (**A**) A schematic diagram of zebrafish 3 dpf larvae with dorsal view. Dotted box depicts the loop structure of BLECs in the optic tectum. Blue lines depict the pronephros. (**B**,**C**) Confocal fluorescence images of the brain optic tectum region with *Tg*(*prox1a:TagRFP*); *Tg*(*fli1a:EGFP*) that labels BLECs and brain vasculatures simultaneously. Control morphant (**B**) and *ccbe1* morphant (**C**) at 3 dpf with BLECs depleted in the brain with intact vasculatures. Scale bar in C = 50 μm. (**D**–**G**) Dorsal view of the larval brains of control (**D**,**E**) and *ccbe1* morphants (**F**,**G**) 4 h after Aβ42 injection at 3 dpf. Red fluorescence represents Aβ42-HiLyte Fluor 647. mAβ42 is seen in BLECs (arrowheads) in the control morphants (**D**) but not in the *ccbe1* morphants (**F**). (**D′**,**E′**,**F′**,**G′**) Confocal images of zebrafish pronephros (dotted lines) after Aβ42 (the same fish with brain images). The robust pronephric accumulation of Aβ42 was detectable in mAβ42-injected control (**D′**), but the reduced pronephric delivery of mAβ42 was observed in *ccbe1* morphants (**F′**) compared to control. oAβ42 injection into both control and *ccbe1* morphants show almost no pronephric accumulation of Aβ42 (**E′**,**G′**). Scale bars = 100 μm. (**H**) Quantification of Aβ42 intensity in the pronephros, normalized by the intensity of non-fluorescent background. (**I**) Quantification of the relative intensity ratio between the pronephros and hindbrain. Statistical significance was determined by ordinary one-way ANOVA with Tukey’s test. HB, hindbrain; MO, morpholino; OT, optic tectum; *n*, independent biological samples or animals. Numbers within bar bottom graphs represent *n*. **, *p* < 0.01; ****, *p* < 0.0001; ns, not significant.

Although BLECs are known to internalize a variety of macromolecules, whether BLECs directly participate in the clearance and drainage of different forms of Aβ42 in the brain has not been functionally validated. To test whether BLECs are necessary for the clearance of Aβ42 in our zebrafish model, we depleted BLECs by knocking down the *ccbe1* gene, encoding a component essential for lymphangiogenesis by processing Vegfc, using a morpholino against *ccbe1* [32]. We confirmed the complete elimination of BLECs in the *ccbe1* morphants with vasculatures grossly intact by using a double transgenic line *Tg*(*prox1a:KalTA4, UAS:TagRFP*); *Tg*(*fli1a:EGFP*) (Figure 4B,C). Upon the cerebroventricular injection of mAβ42 or oAβ42 in the control and *ccbe1* morphants (Figure 4D–G), the depletion of BLECs by *ccbe1* knockdown resulted in a significant reduction in mAβ42 accumulation in the pronephros compared to the control based on the normalized intensity of Aβ42 fluorescence in the pronephros (37.9 ± 3.6% reduction, *p* = 0.0026, ordinary one-way ANOVA with Tukey’s test, *n* = 11 for con MO, *n* = 10 for *ccbe1* MO; Figure 4D′,F′,H). These results suggest that BLECs are required for clearing mAβ42 in the brain through the blood circulatory clearance mechanism. Consistent with the observation that oAβ42 was localized away from BLECs (Figure 3), the accumulation of oAβ42 was almost unchanged irrespective of the presence of BLECs (Figure 4E′,G′,H). The reduced accumulation of mAβ42 in the pronephros upon the depletion of BLECs was also confirmed by comparing Aβ42 fluorescence intensities of the pronephros relative to those of the hindbrain (~35% reduction, *p* < 0.0001, ordinary one-way ANOVA with Tukey’s test, *n* = 11 for con MO, *n* = 10 for *ccbe1* MO; Figure 4D,F,I). The defective pronephric delivery of mAβ42 by the blood circulatory clearance upon BLEC depletion suggests that BLECs participate in clearing mAβ42 from the brain through the blood circulatory routes. On the contrary, the clearance of oAβ42 was not affected in the *ccbe1* morphants, which was consistent with the previous co-localization data (Figure 4E,G–I).

**Figure 5:**
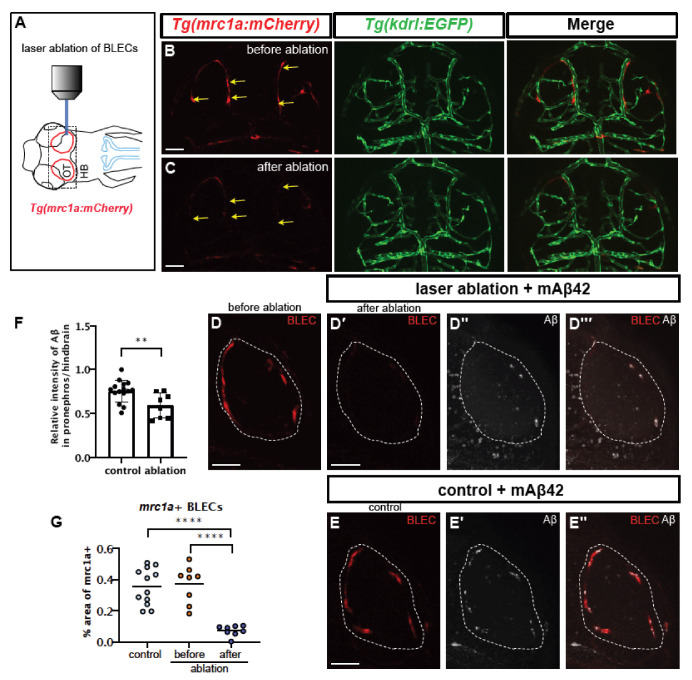
Selective ablation of BLECs decreased internalization of mAβ42 and pronephric accumulation. (**A**) A schematic diagram of the experimental setting. BLEC-specific ablation using the confocal laser. (**B**,**C**) Confocal images of BLECs in the double transgenic *Tg*(*mrc1a:mCherry*); *Tg*(*kdrl:EGFP*) at 3 dpf before laser irradiation (**B**) and after ablation (**C**). Yellow arrows indicate the ablated BLECs. Scale bars = 50 μm. (**D**) Confocal images of *mrc1a*+ BLECs in the loop of the optic before ablation (**D**) and after ablation and mAβ42 injection (**D′**–**D‴**). (**D′**) shows red channel, (**D″**) shows mAβ42 (HiLyte647, white) and (**D‴**) is a merged image. Dotted lines denote the loop of BLECs. (**E**) Confocal images of *mrc1a*+ BLECs with mAβ42 injection (non-ablated control). Scale bars = 50 μm. (**E′**) shows mAβ42 (HiLyte647, white) and (**E’’**) shows merged images of BLECs and mAβ42. (**F**) Quantification of the relative intensity ratio between the pronephros and hindbrain. Data are presented as mean ± SEM. Statistical significance was determined by two-tailed unpaired *t*-test. *n* = 15 for non-ablated control, *n* = 8 for ablated. *p* = 0.0092. (**G**) Quantification of area fraction (%) occupied by *mrc1a+* BLECs. Data are presented as mean ± SEM. Statistical significance was determined by ordinary one-way ANOVA with Tukey’s test. **, *p* < 0.01; ****, *p* < 0.0001.

Since *ccbe1* knockdown may cause the unintended additional depletion of facial and trunk lymphatics, we performed a more fine-tuned cell ablation strategy specific for BLECs using direct laser ablation. The laser ablation protocol, using a laser-scanning confocal microscope equipped with the common 405 nm laser [33], resulted in the specific ablation of BLECs (Figure 5A–C) without any obvious defect in the cerebrovasculature revealed by *Tg*(*kdrl:EGFP*) (Figure 5B,C). The laser irradiation resulted in a significant reduction in the number of *mrc1a:mCherry*-positive cells in the brain region (~79.74% reduction in *mrc1a*+ BLECs compared to the number before ablation) (Figure 5G). To confirm the function of BLECs in Aβ clearance after specific ablation, mAβ42 was injected into the ventricle after BLEC ablation and the pronephric accumulation of Aβ42 was measured (Figure 5D–F). The BLEC ablation using the laser resulted in a significant reduction in mAβ42 localized in the BLECs (Figure 5D,D‴) compared to the control (Figure 5E,E′). Importantly, the mAβ42 drainage to the pronephros was significantly reduced (~21% reduction, *p* = 0.0092, two-tailed unpaired *t* test, *n* = 15 for control, *n* = 8 for ablation; the combined results for three experiments with the identical experimental settings) (Figure 5F), confirming a specific requirement of BLECs in the mAβ42 clearance from the brain.

### 2.5. Mannan Administration Reduces mAβ42 Internalization by BLECs and Peripheral Transport

**Figure 6:**
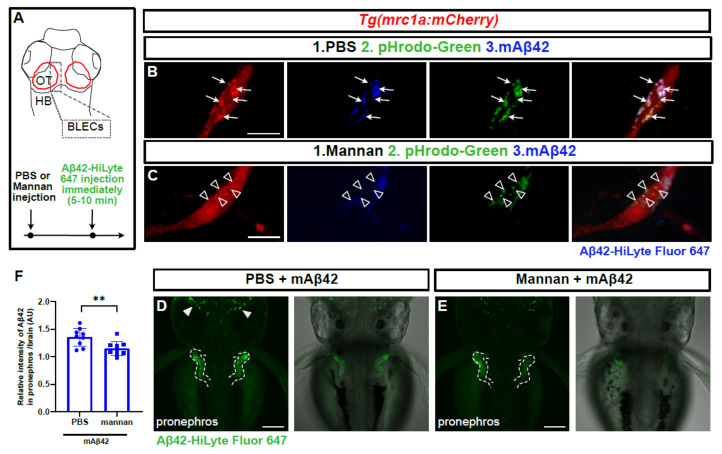
Mannan administration reduces uptake of mAβ42 by BLECs and peripheral transport. (**A**) A schematic diagram of dorsal view of the 3 dpf larval brain and the experimental setup. Dotted gray box denotes the region of interest. (**B**,**C**) Confocal images of *mrc1a:mCherry*+ BLECs co-injected with pHrodoGreen and mAβ42 (HiLyte Fluor 647). Arrows indicate colocalization of pHrodoGreen and mAβ42 (**B**). Empty arrowheads show that the mannan administration interferes with colocalization of pHrodoGreen and mAβ42. Data are representative of at least three independent experiments. Scale bars = 10 μm. (**D**,**E**) Confocal images of the zebrafish pronephros after mAβ42 injection. Dotted lines depict the pronephros structure. Arrowheads indicate the accumulation of mAβ42. Mannan treatment prior to mAβ42 injection (**E**) resulted in a reduced pronephric accumulation compared to PBS control (**D**). Scale bars = 100 μm. (**F**) Quantification of the relative ratio of the intensity between the pronephros and brain. Statistical significance was determined by two-tailed unpaired *t*-test. *p* = 0.0092. Data are presented as mean ± SEM. *n* = 9 per group. Data are representative of at least two independent experiments. **, *p* < 0.01.

BLECs take up macromolecules from the CSF in a Mannose receptor 1a (Mrc1a)-dependent manner; this process is abrogated by the treatment of mannan, a known competitive agonist of Mrc1a [16]. We reasoned that the internalization of mAβ42 into BLECs may also be Mrc1a-dependent, and thus affected by mannan treatment. We tested the BLEC function in clearing mAβ42 pharmacologically with a minimal effect on undesirable vascular function at a systemic level. We introduced mannan and pHrodo Green into the brain ventricle immediately followed by an Aβ42 injection within 5–10 min and examined whether the mAβ42 uptake into BLECs and its clearance were affected. Mannan administration showed grossly similar localization patterns of mAβ42 in BLECs to PBS administration (Figure 6B,C). However, the high-resolution confocal imaging revealed that the mAβ42 fluorescence observed in BLECs upon mannan treatment accumulated mostly in the membrane surfaces and did not co-localize with the pHrodo marker suggesting the failure of the internalization of mAβ42 into the cytoplasmic components of BLECs upon mannan treatment (Figure 6C). Consistent with this observation, mannan treatment also significantly reduced the pronephric accumulation of mAβ42 compared to PBS treatment (~15% reduction, *p* = 0.0092, Figure 6D–F), suggesting that BLECs were implicated in mAβ42 clearance, partly via the Mrc1a-dependent internalization of mAβ42. Taken together, the depletion of BLECs by a genetic knockdown and laser ablation, as well as the inhibition of BLECs, functions by a pharmacological method and shows a significant reduction in the pronephric accumulation of mAβ42 in the zebrafish Aβ clearance model that we developed, supporting the notion that that BLECs are one of the key components of the clearance system for Aβ42 within the brain, selectively internalizing mAβ42, but not oAβ42, and removing it through the blood circulatory clearance route.

### 2.6. An Aβ42 Aggregation Inhibitor Promotes oAβ42 Localization into BLECs and the Peripheral Transport

**Figure 7:**
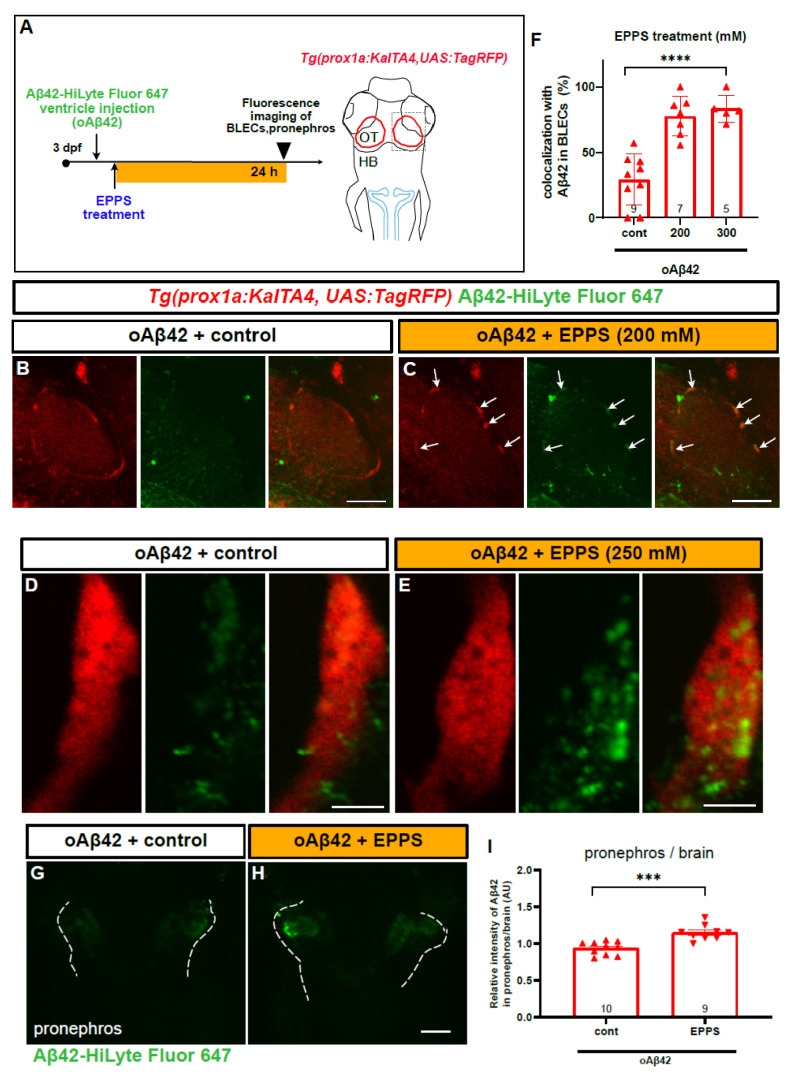
EPPS treatment enhances the BLEC localization and pronephric transport of oAβ42. (**A**) A schematic diagram of EPPS treatment after oAβ42 (HiLyte-Fluor 647) injection. Gray dotted box depicts the region of interest and blue lines indicate the pronephros. (**B**–**E**) Confocal images of *prox1a:RFP+* BLECs in the loop of the optic tectum of oAβ42-injected larval brain with control (**B**,**D**) and EPPS treatment (**C**,**E**). (**B**,**C**) Co-localization of BLECs with oAβ42 increased upon EPPS treatment (arrows) in **c** compared to control (**B**). Scale bars in (**B**,**C**) = 50 μm. (**D**,**E**) Confocal images of *prox1a:RFP+* BLECs with EPPS treatment (250 mM, **e**) after oAβ42 injection with high magnification revealed the internalized oAβ42 in BLECs compared to control (**D**). Scale bars in (**D**,**E**) = 5 μm. (**F**) Quantification of oAβ42 co-localization in BLECs (%) upon EPPS treatment. Statistical significance was determined by ordinary one-way ANOVA with Tukey’s test. (**G**,**H**) Confocal images of the pronephros (white dotted lines) injected with oAβ42 in control (**G**) and with EPPS treatment for 24 h (**H**). (**I**) Quantification of the relative ratio of the intensity between the pronephros and brain. Two-tailed unpaired *t*-test, *p* = 0.0002. Data are presented as mean ± SEM. Data are representative of at least two independent experiments. Numbers within bar bottom graphs represent *n*. ***, *p* < 0.001; ****, *p* < 0.0001.

In order to further confirm the functionality of BLECs in mAβ42 clearance and assess the utility of our zebrafish model for validating an AD drug candidate, we tested the efficacy of the small molecule EPPS in Aβ42 clearance, which is known to convert aggregated Aβ into monomers and remove Aβ plaque and oligomers from the brain of an AD mouse model [34]. We explored whether EPPS treatment induced the localization change of oAβ42 into BLECs and facilitated their clearance out of the brain into the pronephros, presumably by converting oAβ42 into mAβ42. First, oAβ42 (HiLyte Fluor 647) was injected into the brain., immediately followed by an EPPS incubation for the next 24 h and then the imaging of the BLEC loop from the optic tectum region with confocal microscopy (Figure 7A). Upon EPPS treatment, the co-localization of oAβ42 with BLECs was significantly increased in a dose-dependent manner compared to the vehicle-treated control (arrows in Figure 7B,C, F). High-resolution confocal imaging revealed the increased co-localization of oAβ42 in the internal components of the *prox1a*+ BLECs upon EPPS treatment (Figure 7D,E). We also examined whether the EPPS treatment enhanced the pronephric accumulation of oAβ42. Consistent with the increased co-localization of oAβ42 with BLECs, the EPPS treatment of oAβ42-injected larvae significantly increased the fluorescence intensity in the pronephros compared to the untreated oAβ42-injected control (Figure 7G,H). The enhanced accumulation of oAβ42 in the pronephros with EPPS was verified by comparing the Aβ42 fluorescence intensities of the pronephros relative to those of the brain (Figure 7I, ~23% increase, *p* = 0.0002, two-tailed unpaired *t* test, *n* = 10 for control, *n* = 9 for EPPS 250 mM). As expected, EPPS treatment in mAβ42 did not induce significant differences in the co-localization with BLECs, with a slight increase in the pronephric accumulation compared to the untreated control (Figure S8B–G). These data revealed the Aβ42 clearance process of the candidate AD drug EPPS that was known to disaggregate oAβ42 and recover behavior defects in mouse models [34] by visualizing the increased relocalization of the disaggregated oAβ42 into BLECs for clearance. Therefore, improving the functionality of BLECs may be a novel therapeutic approach for treating AD by enhancing the efficient removal of mAβ42 converted from Aβ42 aggregates and lowering Aβ toxicity in combination with Aβ42 disaggregating agents.

## 3. Discussion

The initial accumulation and defective clearance of Aβ are considered as early phases of AD before clinical symptoms appear [35]. In the current study, we established an in vivo zebrafish model to investigate the brain clearance system regarding the accumulation of Aβ42, which is one of the main Aβ isoforms and displays a strong neurotoxicity. In this model, we visualized and monitored the real-time, in vivo localization and clearance of Aβ42 in the brain, by injecting fluorescently labeled Aβ42 peptides into the ventricle of the zebrafish larval brain, revealing the differential clearance dynamics of Aβ42 from the brain depending on its aggregation status (i.e., mAβ42 and oAβ42). We found that this differential clearance was mediated by BLECs, a recently re-identified lymphatic cell population in the brain. BLECs participated in the selective internalization of Aβ42 monomers, but not Aβ42 oligomers, and cleared them through the blood circulatory clearance pathway, resulting in the accumulation of mAβ42 in the pronephros for excretion. The role of BLECs in clearing mAβ42 was functionally validated by genetic depletion, pharmacological inhibition, or specific ablation using laser-targeting BLECs that prevented the efficient clearance of mAβ42 from the brain and, conversely, by the promoted clearance of oAβ42 with EPPS treatment, a protein-disaggregating agent that facilitates the conversion of oAβ42 to mAβ42. Taken together, our data suggest that BLECs are one of the main components in Aβ42 homeostasis in the brain clearance system. We propose that the dysfunction of BLECs is a causative factor in AD pathogenesis and the recovery and/or enhancement of BLEC function, especially in combination with an Aβ disaggregation strategy, as a legitimate therapeutic and diagnostic approach against pathological Aβ42 accumulation.

### 3.1. Cerebroventricular Injection of Aβ for Modeling Alzheimer’s Disease in Zebrafish

Previous studies utilized direct Aβ injection methodology to generate AD-like symptoms in mouse models because of the rapid induction of phenotypes and for economic reasons [20,36]. Similarly, a few zebrafish Aβ injection models were established to validate a chaperone-gold nanoparticle as a novel AD drug that mitigated Aβ toxicity and AD-like behavioral defects in the larval and adult stages, to identify upregulated intereleukin-4 that activated neural stem cell proliferation for regeneration after Aβ-induced neuronal death in the adult brain, and to study the function of Aβ oligomers and distinct molecular pathways in the sleep/wake regulation with a zebrafish larval model [37,38,39]. All of these studies indicated the applicability of the direct Aβ injection strategy in zebrafish AD modeling. In line with these studies, we successfully established a cerebroventricular Aβ42 injection model for the early larval stages (3~4 dpf), when the externally located zebrafish larval brain is transparent and accessible. This allows a direct in vivo live imaging with a high resolution and readily amenable genetic/chemical manipulations via a direct injection in a fast and cost-effective way. With our Aβ42 injection model, we addressed for the first time the mechanistic aspects of Aβ42 clearance in the brain depending on their aggregation status by the selective nature of BLECs, an under-studied lymphatic cell population in the brain. Our model provides a useful platform to investigate the underlying mechanisms of Aβ dynamics in the brain, as well as to validate candidate modifiers (genes and chemicals) of AD-like phenotypes in vivo in great detail.

### 3.2. BLEC Is a Novel Component of the Brain Clearance System to Remove mAβ42

The proteins in the brain can be removed by various clearance mechanisms [6]. Generally, extracellular proteins such as Aβ in the CSF can be cleared by absorption into the circulatory system, including via the blood–CSF barrier (BCSFB) or from the meningeal lymphatic vessels to the cervical lymph node [6]. The latest findings of meningeal lymphatic vessels in mammals are broadening our understanding of how the brain maintains a fluid balance and removes cellular waste; in mammals, the meningeal lymphatic vessels, particularly in the basal part of the skull (the basal meningeal lymphatic vessels), drain CSF and clear macromolecules with their specialized morphologies [13]. The CSF in the basal meningeal lymphatic vessels drains into the cervical lymph nodes, shown by macromolecular tracers [13]. The ablation of meningeal lymphatic vessels, ageing, or a disease context such as in an AD mouse model, impaired these effluxes [12,13]. The solutes in the cervical lymph nodes are likely to be transported into the blood and degraded in the liver or kidney [6,24]. Consistent with the mammalian system, zebrafish were recently found to possess a meningeal lymphatic network with draining function at juvenile and adult stages [40], although the exact drainage spots for meningeal lymphatics, whether the CSF directly drains into the blood vessel or other lymphatic network, or whether an undiscovered cervical lymph node exists as in mammals, remain to be addressed. In the current study, we focused on the BLECs as one of the components of the brain clearance system in zebrafish. Although BLECs have a lymphatic identity, they do not form lumen and are loosely connected with the surrounding cerebrovasculature, unlike meningeal lymphatic vessels. In addition, their locations in the meninges are quite distinct because BLECs are localized in the deeper layer of the mouse brain (leptomeninges, the arachnoid, and the pia mater) and adjacent to the cerebral blood vessels [13,14,15], whereas meningeal lymphatic vessels are located in the outer layer of meninges both in mouse (dura mater) and zebrafish (below the skull) [17,40]. Since BLECs are a sort of cell population clearly distinct from meningeal lymphatic vessels in many aspects as described above, BLECs may not be directly implicated in the drainage of the general lymphatic system in the brain at present. As our current studies reveal an Aβ clearance pathway mediated by BLECs from the brain to the peripheral tissues, further study is required to clarify the functional linkage between BLECs and meningeal lymphatics in the juvenile or adult stages of zebrafish.

BLECs were originally discovered as fluorescent granule perithelial cells (FGPs) in mammals in the 1980s [41] and recently re-identified in zebrafish, with a diverse naming as brain/mural lymphatic endothelial cells (BLECs/muLECs), FGPs, or Mato cells; they have unusual characteristics, such as a macrophage-like morphology, lymphatic lineage, and unique locations in the perivascular area [14,15,16]. Since there are still no specific markers for the BLECs, it is currently difficult to distinguish the BLECs from the CNS macrophages, which also express the lymphatic markers such as Mrc1 and Lyve1 (lymphatic vessel endothelial hyaluronic acid receptor 1) [42]. Thus, the presence and identity of BLECs in mammals still remain controversial among researchers, as well as whether the LLECs (murine BLECs) are a distinct cell type from other cells such as perivascular macrophages or FGPs, warranting further comprehensive studies in both mammals and zebrafish with in-depth comparative approaches based on detailed anatomical, molecular, and transcriptomic analyses.

Notably, FGPs (also called Mato cells) in mouse and rat models are reported to be implicated in the pathogenesis of cerebral amyloid angiopathy (CAA), in which the aberrant deposition of Aβ in blood vessel walls are frequently detected in the leptomeningeal and cortical arteries [42,43]. Several studies suggested that the uptake capacity of FGPs in the cerebral vessels is affected by aging, using electron microscope observations [41,44], while other correlative studies suggested the correlation of the vesicular uptake of perivascular macrophages with the onset of AD [41,44,45]. Furthermore, the functional contribution of perivascular macrophages to CAA was suggested based on the finding that the depletion of perivascular macrophages in an AD mouse model resulted in Aβ deposition along the vasculature [46], although the distinction between the macrophages and FGPs is not clear in this study. As there is still no direct evidence or association of BLECs with mammalian diseases such as AD or CAA, our findings on the BLECs-mediated blood circulatory clearance of Aβ may be represented as indirect evidence for the implication of BLECs in CAA, regardless of whether BLECs are unique cell populations distinct from FGPs, as Shibata-Germanos suggested [17], or one of the subpopulations of FGPs [15,40].

### 3.3. BLECs Selectively Clear Monomeric Aβ from Brain with a Blood Circulatory Route

Sporadic AD patients exhibit an impairment in Aβ peptide clearance rather than production [3,47]. In addition, several risk genes associated with sporadic AD are also thought to affect Aβ clearance [48,49]. These findings illustrate the importance of understanding and targeting the brain clearance system to develop an efficacious AD therapy. The decreased clearance of Aβ results in the accumulation of various isoforms of Aβ monomers and their aggregation into oligomers, fibrils, and plaques in the central nervous system [47]. Despite the pathological relevance and toxicity of oAβ, little is known about how it is cleared from the brain. A handful of AD mouse model studies suggested that oAβ may be less efficiently cleared from the brain due to its nature of resisting enzymatic degradation in the brain or its increased molecular size preventing glymphatic transport [10,22]. In the current study, the comparison of monomeric and oligomeric Aβ42 clearance using our zebrafish model revealed that mAβ42 readily interacted with and accumulated in BLECs, a less-characterized clearance component in the brain used for efficient clearance. On the contrary, oAβ42 rarely accumulated in BLECs, which contributed to its preferential accumulation in the brain.

Given the role of BLECs in the internalization and clearance of macromolecules in the brain, we hypothesized that BLECs were implicated in the Aβ42 clearance from the brain and aimed to elucidate their precise function in the clearance of differently aggregated forms of Aβ42 peptides. Our data suggest that BLECs are one of the critical components for Aβ clearance as they selectively internalize mAβ42. Distinct from the recently discovered meningeal lymphatic vessels in the zebrafish brain after the juvenile stages (initially developed after 9~10 dpf [40]), BLECs appear to play a role in drainage function, at least during the larval stages of zebrafish. This conclusion was based on the findings that (i) BLECs readily internalized the ventricle-injected mAβ42 and then delivered it to the periphery as shown in the accumulation in the pronephros (Figure 2 and Figure 3), (ii) BLECs-accumulated mAβ42 fluctuated dynamically and disappeared in real time in vivo (Figure 3 and Figure S7; Supplementary Video S1), and (iii) abrogating BLEC function by the depletion with *ccbe1* knockdown, laser ablation or mannan delivery into the brain prior to mAβ42 injection resulted in the failure of Aβ clearance to the periphery (Figure 4, Figure 5 and Figure 6).

We also showed that the pronephric accumulation of mAβ42 was based on the blood circulatory route with propranolol and *tnnt2a* morphants (Figure 2). Although the genetic depletion of BLECs by *ccbe1* MO resulted in a reduced pronephric accumulation of mAβ42 up to ~35% decline (Figure 4), the specific laser ablation of BLECs showed a ~21% reduction in the pronephric delivery (Figure 5). This may suggest that BLECs are only partly responsible for the blood circulatory clearance of mAβ42 and, although additional clearance routes exist, we cannot currently exclude the possibility of the incomplete ablation of BLECs.

The exact clearance mechanisms of BLECs in clearing mAβ42 via the blood circulatory route is still unclear. It was reported that Aβ-bearing perivascular monocytes played a role in Aβ clearance via crawling veins and circulated back into the blood with Aβ [50], suggesting that the Aβ-scavenging cells such as monocytes, not only degrade Aβ intracellularly, but also contribute to blood circulatory clearance. It is possible that BLECs may clear mAβ42 in a similar fashion via the interaction of nearby vasculatures, in a similar way to perivascular monocytes, although we could not observe such cellular movement of BLECs in our time-lapse imaging (Figure S7; Supplementary Video S1). Alternatively, it may be also feasible that the extracellular vesicles and exosomes may be released and taken up between the BLECs and endothelial cells, similar to the cell-to-cell communicating process known to occur among diverse cell types composing the neurovascular unit [51].

Interestingly, the injected oAβ42 (with predicted sizes in the aqueous buffer of 100–300 kDa [4]), failed to be internalized by BLECs (Figure 3), despite the fact that BLECs have the capability of internalizing macromolecules of up to 500 kDa [16]. This discrepancy may be due to the distinct characteristics of Aβ oligomers, such as their unique and various structural natures, rather than their size per se [52]. This failure of oAβ42 clearance was significantly reversed by treating the BLECs with the Aβ-disaggregating agent EPPS with an increased pronephric delivery (Figure 7), thereby illustrating the therapeutic benefit of an Aβ-disaggregating agent on toxic oAβ clearance. Curiously, Aβ disaggregated by EPPS may re-form Aβ aggregates in the absence of a proper clearance system or immune responses to remove the disaggregated Aβ, as shown in an AFM study with EPPS [53]. Thus, the improvement of the brain clearance system via the enhanced functionality of BLECs coupled with Aβ disaggregation would provide an efficient therapeutic strategy to prevent AD pathogenesis by reducing the accumulation of the toxic Aβ oligomers in the brain.

## 4. Materials and Methods

### 4.1. Animals

Zebrafish (*Danio rerio*) AB strain and transgenic lines were kept at 28.5 °C in 14 h light and 10 h dark cycle. The lymphatic system was visualized using *Tg*(*prox1a^BAC^: KalTA4-4xUAS-E1b:uncTagRFP*) [29] (herein denoted as *Tg(prox1a:KalTA4, UAS:TagRFP*; kindly provided by Prof. Suk-Won Jin). Blood vessels were visualized with *Tg*(*kdrl:EGFP*) and *Tg*(*fli1a:EGFP*). To generate BLEC- and lymphatic vasculature-specific transgenic zebrafish, *Tg*(*mrc1a:mCherry*) was newly generated according to a previous study [54]. Briefly, 2.1 kb genomic sequence upstream from the transcription start site of zebrafish *mrc1a* gene, combined with 0.3 kb enhancer sequence located in intron 20, was cloned into pTol2 vector using Gibson assembly (New England Biolabs, Ipswich, MA, USA). Tol2-based *mrc1a:mCherry* expression construct was prepared using plasmid endotoxin free mini kit (Geneaid, New Taipei City, Taiwan) following the manufacturer’s protocol. To generate transgenic zebrafish, one-cell-stage eggs were injected with approximately 1~2 nL of a DNA/RNA solution containing 25 ng/μL Tol2 transposase mRNA and 20 ng/μL Tol2-based *Mrc1a:mCherry* expression construct [55]. The embryos showing targeted tissue-specific fluorescence were screened and raised to the adulthood. The F0 adult fish were crossed to WT fish for analyzing germline transmission by screening the offspring under the M205FCA microscope (Leica, Wetzlar, Germany). To avoid pigmentation, embryos were treated with 0.003% 1-phenyl-2-thiourea (PTU) (Sigma, St. Louis, MO, USA) in E3 egg water (5 mM NaCl, 0.17 mM KCl, 0.33 mM CaCl_2_, and 0.33 mM MgSO_4_). All animal experiments were carried out in accordance with permits and guidelines of the Korea Research Institute of Bioscience and Biotechnology (KRIBB) and approved by KRIBB-IACUC (approval number: KRIBB-AEC-20200).

### 4.2. Aβ preparation and Ventricle Microinjection

Fluorescence (HiLyte Fluor 488, 555, 647)-labeled amyloid-β (1-42) (DAEFRHDSGYEVHHQKLVFFAEDVGSNKGAIIGLMVGGVVIA) peptides were purchased from AnaSpec (Fremont, CA, USA). Aβ was diluted with DMSO at 2 μg μL^−1^ and stored at −80 °C deep freezer before use. HiLyte-conjugated Aβ (1-42) stock solution was diluted in 1XPBS (phosphate-buffered saline) (1:9 *v*/*v*) and the oligomeric form of Aβ was prepared by incubating at 37 °C for 3 days as previously described [20]. The monomeric form of Aβ was immediately used without incubation. Microinjections were carried out with a Pneumatic PicoPump (World Precision Instruments, Sarasota, FL, USA) and capillary needles prepared by a Micropipette puller (Sutter instrument, Novato, CA, USA). For ventricle injection, 3 dpf larvae were anesthetized with tricaine (Sigma, St. Louis, MO, USA) and placed in 1% low-melting agarose confocal dish and injected with the total volume of 1–2 nL of the Aβ solution. Trimmed needles were inserted into the ventricular space between the optic tectum and hindbrain, in order to not penetrate into deep brain tissues.

### 4.3. Confocal Microscopic Analyses for Aβ Clearance and Quantification

For live confocal imaging of zebrafish larvae, 3 dpf or 4 dpf larvae were anesthetized with tricaine and mounted in 1% low-melting agarose on confocal dishes. Then, the agarose was covered with tricaine solution. Confocal Z step size was at 4~5 μm with 20× objective lens and Z projections were generated by stacking 25 optical slices for the brain region and 20 optical slices for the pronephros region, using FV1000 confocal microscope (Olympus, Tokyo, Japan) or Zeiss LSM800 microscope (Carl Zeiss, Oberkochen, Germany). Z projection images of injected Aβ42 fluorescence in the brain were converted to thresholded (pixel intensity, 40-255) images, and areas of particles in calibrated square unit (512 × 512 pixels) were measured as a percentage using Image J (National Institute of Health, Bethesda, Maryland, MD, USA, https://imagej.nih.gov/ij/ (accessed on 8 October 2021), v1.52, accessed on 1 August 2021). Clearance index was determined by following formula (“reduction rate” (%) = particle area of fluorescence at 5 hpi-particle area of fluorescence in 24 hpi/particle area of fluorescence in 5 hpi). The Aβ42 fluorescence intensity of the pronephros was measured in the region of interest and analyzed using Image J. The Aβ42 fluorescence intensity of each pronephros was measured and normalized by background signals in non-fluorescence area and presented as fold differences using Image J. The relative Aβ42 intensity of each pronephros to the brain was also calculated by dividing the Aβ42 intensity of the pronephros by the mean fluorescence intensity of each brain. Quantification of Aβ42 co-localization with BLECs was presented as percentages of the number of Aβ42 positive BLECs over the whole number of BLECs.

### 4.4. pHrodoGreen Injection

To validate the internalization of BLECs, an endocytic marker, pHrodoGreen dextran, 10 kDa (ThermoFischer, Waltham, MA, USA, P35368) was prepared in PBS at 2 mg/mL concentration and injected into the brain ventricle of 3 dpf zebrafish larvae [16,18] before mAβ42 or oAβ42 was introduced. In the mannan experiment, pHrodoGreen was injected after PBS or mannan administration.

### 4.5. Genetic and Pharmacological Inhibition of Heartbeat

To reduce the heartbeat in mAβ42-injected zebrafish larvae, 100 μM of propranolol, a β-blocker (Sigma, St. Louis, MO, USA), was incubated for 4 h at 3 dpf larvae or 200 μM of *tnnt2a* morpholino (*tnnt2a* ATG MO 5′-CAT GTT TGC TCT GAT CTG ACA CGC A) was injected at one cell stage [27]. The heartbeat was counted for 10 s and extrapolated. Pronephros imaging was conducted in 1% low-melting agarose containing the propranolol.

### 4.6. Intravenous Injection of Endocytic Tracer

To verify vascular route of mAβ42 and visualize the pronephros in our model, intravenous injection of endocytic tracer was performed as previously described [28]. 10 kDa dextran labelled with Alexa Fluor 647 (ThermoFisher, Waltham, MA, USA, D22914) was prepared in PBS at 2 mg/mL final concentration and injected into caudal vein of 3 dpf zebrafish larvae into the brain ventricle of which mAβ42 or oAβ42 was injected at 3 dpf. For sectioning of the pronephros region, larvae were mounted in cryosectioning molds, frozen on dry ice, and sectioned using a Leica CM1860 cryostat (Leica, Wetzlar, Germany).

### 4.7. Genetic and Pharmacological Inhibition of Brain Lymphatic Endothelial Cells

Morpholino oligonucleotides (*ccbe1* ATG MO 5′-CGG GTA GAT CAT TTC AGA CAC TCT G-3′; control MO 5′-CCT CTT ACC TCA GTT ACA ATT TAT A-3′) [32] were purchased from Gene Tools (Philomath, OR, USA) and diluted in nuclease free water to make 1 mM stock. The prepared morpholinos were injected at one to four cell stages at a concentration of 500 μM with 0.25% phenol red (Sigma, St. Louis, MO, USA) using a Pneumatic PicoPump. To interfere with the mannose receptor-dependent endocytosis of BLECs, mannan (Sigma, St. Louis, MO, USA) (50 mg/mL, in PBS) was injected into the larval brain ventricle 10 min prior to Aβ42 injection.

### 4.8. Laser Ablation of Brain Lymphatic Endothelial Cells

Laser ablation of BLECs was performed according to a previous study based on confocal microscope laser ablation with several modifications [33]. *Tg(mrc1a:mCherry)* larvae were raised in PTU until 3 dpf and mounted in the 1% low-melting agarose. *mrc1a:mCherry*-positive cells in the zebrafish brain were ablated using a 405 nm laser of confocal microscope (FV1000 confocal microscope, Olympus, Tokyo, Japan). Briefly, using the 60x oil immersion objective a cell body of BLECs was positioned in the center for ablation. The laser power of 405 nm was adjusted to 100% and the scanning mode was activated during 90 s. Because the whole BLEC ablation process for a larva takes approximately 40 min–1 h, BLECs of only two to three samples could be ablated for a single set of experiments due to time constraints, the statistical significance was determined by sum of three independent experiments performed with the identical experimental settings. Larvae were imaged first before ablation, followed by mAβ42 injection, and then another round of imaging for pronephric accumulation measurement after ablation.

### 4.9. EPPS Treatment

For Aβ disaggregation, EPPS [4-(2-hydroxyethyl)-1-piperazinepropanesulfonic acid] was purchased (Sigma, St. Louis, MO, USA) [34]. EPPS solution was prepared as 1 M stock by dissolving with distilled water. EPPS was added to PTU E3 egg water up to 200–300 mM and incubated for 24 h after Aβ42 injection. EPPS-treated larvae were mounted in the low-melting agarose for imaging.

### 4.10. Statistical Analysis

All statistical calculations and analyses were performed using Prism (Version 8.4.2, GraphPad, San Diego, CA, USA). Statistics were performed using an unpaired *t* test or ordinary one-way ANOVA. Data were represented as mean ± standard error of the mean (±SEM).

## Data Availability

The data presented in this study are available on request from the corresponding author.

## Supplementary Materials

The following are available online at https://www.mdpi.com/article/10.3390/ijms222111883/s1.

## Abbreviations

Aβ	amyloid beta
AD	Alzheimer’s disease
AFM	atomic force microscopy
BBB	blood–brain barrier
BLEC	brain lymphatic endothelial cells
CSF	cerebrospinal fluid
FGPs	fluorescent granule perithelial cells
ISF	interstitial fluid
mAβ42	monomeric Aβ42
oAβ42	oligomeric Aβ42
