# Supplementary material for: Differential Clearance of Aβ Species from the Brain by Brain Lymphatic Endothelial Cells in Zebrafish
Source: Int J Mol Sci. 2021 Nov 2;22(21):11883. doi: 10.3390/ijms222111883 (PMC8584359; doi:10.3390/ijms222111883)
Supplement: Supplementary file 1 [file ijms-22-11883-s001.zip › Supplementary Information_JSL.pdf]

Supplementary Information

Supplement to "Differential Clearance of Aβ species from the Brain by Brain Lymphatic Endothelial Cells in Zebrafish" by *Jeong et al.*

Table of Contents

Supplementary Materials and Methods ………………………………..2

Supplementary Figures…………………………………4

Supplementary Materials and Methods

Atomic force microscopy (AFM) analysis of mAβ42 and oAβ42

The Aβ42 solution was dropped onto the mica substrate and deposited overnight. After rinsing the substrate for several times with distilled water, the surface topographic analysis of Aβ42 was performed by Atomic Force Microscopy (AFM, XE-100, Park Systems, Suwon, Republic of Korea). The measurements were performed in the non-contact mode using a silicon probe at a scan rate of 0.8 Hz to a 1 μm^2 area.

Immunofluorescence

Whole-mount immunostaining of Aβ42-injected larvae was performed as previously described (Randlett et al., 2015). Briefly, 3 dpf or 4 dpf larvae were fixed in 4% paraformaldehyde diluted in 1X PBS with 0.25% Triton (PBST) at 4 °C overnight. Larvae were washed with PBST three times and then incubated in 150 mM Tris-HCl (pH 9.0) at 70 °C for 20 minutes to retrieve antigens. Following quick wash with PBST, larvae were treated with 0.05% Trypsin-EDTA for 45 minutes on ice. The larvae were rinsed with PBST, incubated with a blocking buffer (PBST with 2% normal goat serum, 1% bovine serum albumin, and 1% dimethyl sulfoxide) for one hour. Primary antibodies were incubated in the blocking buffer at 4 °C overnight. Larvae were washed three times for 15 minutes with PBST and then incubated with secondary antibodies at 4 °C overnight. Next day, they were washed with PBST, mounted, and imaged. Primary antibodies used were 6E10 (1:500, anti-amyloid β 1-16 antibody, Biolegend) and 4G8 (1:500, anti-amyloid β 17-24 antibody, Biolegend). Secondary antibodies were Goat anti-Mouse IgG Secondary Antibody, Alexa Fluor Plus 555 (1:500, Thermofischer).

Acridine Orange staining

To visualize cell death after brain ventricle injection, 3 dpf larvae were incubated in 10 μg ml^{-1} Acridine Orange (Sigma) in E3 egg water for one hour in the dark. Then larvae were washed with E3 egg water three times and mounted in low-melting agarose for fluorescence imaging.

Time-lapse imaging

Confocal time-lapse imaging of HiLyte-fluor Aβ42 injected larvae at 3 dpf was performed by embedding larvae in 1% low melting agarose using a 20X objective lens of FV1000 confocal microscope (Olympus). Z projections were generated by stacking 25 optical slices for the brain region starting at 4 hpi and continuing one hour (10 minutes per frame, 7 frame). Image brightness and contrast were adjusted in ImageJ (National Institute of Health) and image stacks were exported as AVI format with 3 frame per seconds (fps).

Supplementary references

Randlett O, Wee CL, Naumann EA, Nnaemeka O, Schoppik D, Fitzgerald JE, et al. Whole-brain activity mapping onto a zebrafish brain atlas. Nat Methods. 2015; 12: 1039-46.

Supplementary Figures

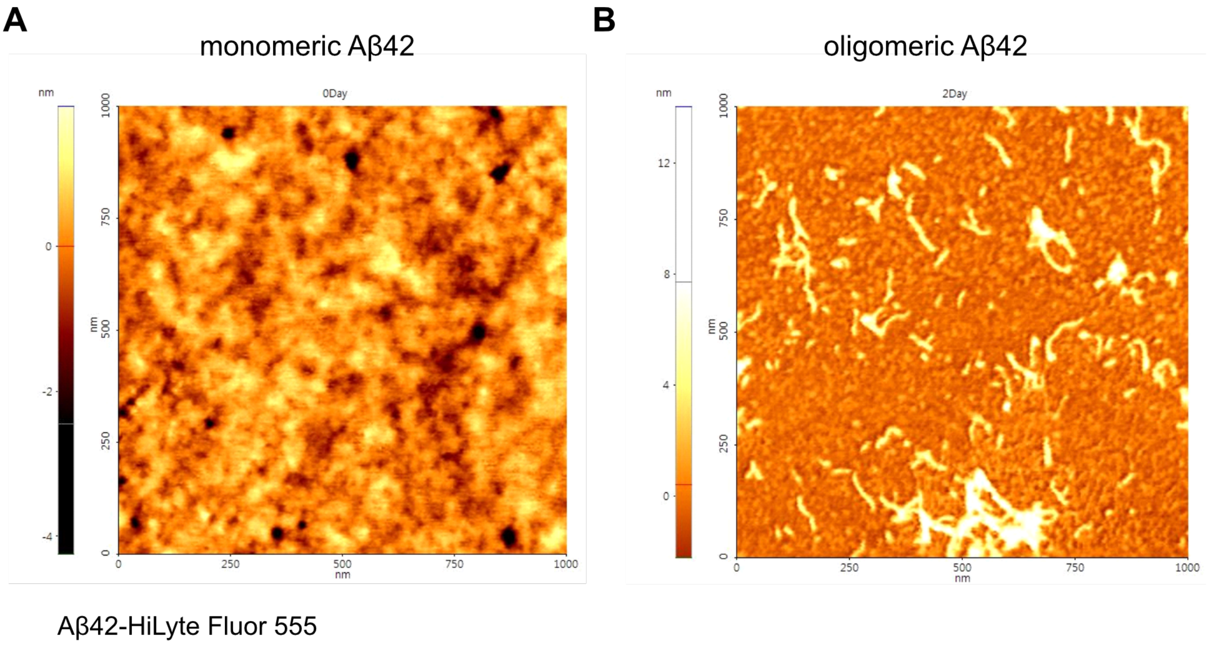

Figure S1. Oligomeric Aβ42 peptides form fibrillary structures while monomeric Aβ42 peptides are globular. (A) Monomeric Aβ42. (B) Oligomeric Aβ42.

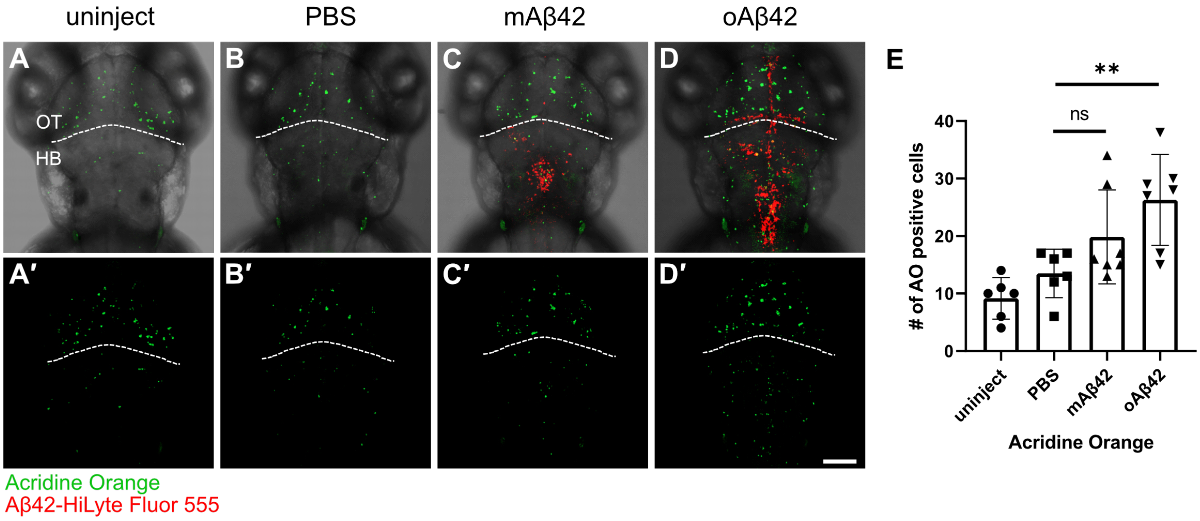

Figure S2. Oligomeric Aβ42 peptides are more toxic to cells in the hindbrain of zebrafish larvae. (A-D) Merged images of 3-day larvae injected PBS (B), mAβ42 (C), or oAβ42 (D) together with acridine orange (AO) staining and brightfield background. (A′-D′) Images of AO staining only. (E) Quantification of AO-positive cells in the hindbrain. AO, acridine orange; OT, optic tectum; HB, hindbrain; ns, not significant. **, $p < 0.005$. Scale bar = 100 μm

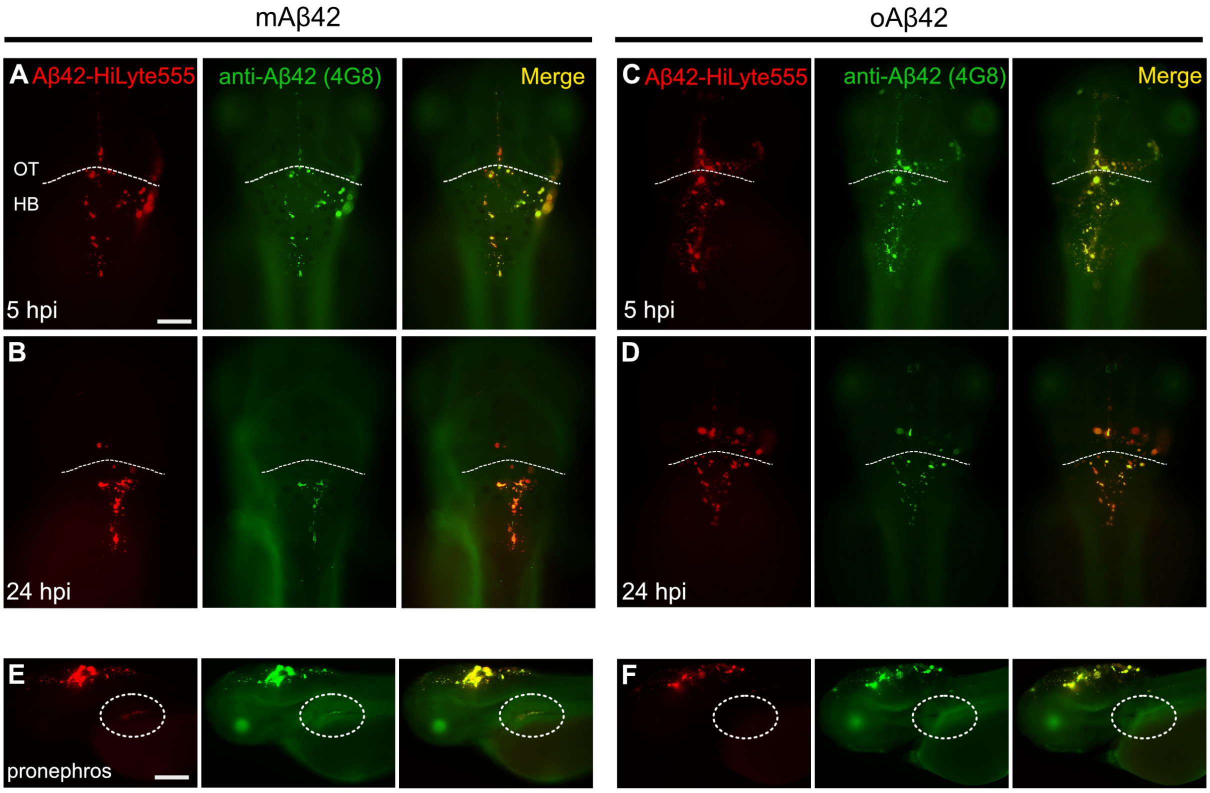

Figure S3. The fluorescence of HiLyte fluorophore-labeled Aβ42 coincides with anti-pan-Aβ42 antibody. (A-D) Fluorescence images of the whole-mount immunostained 3 dpf larvae with anti-pan-Aβ42 antibody (4G8, green) injected with mAβ42 (A, B) and oAβ42 (C, D), dorsal view. Co-localized immunostaining of the Aβ antibody and the fluorescence of HiLyte-labeled Aβ42 at 5 hpi (A, C) and 24 hpi (B, D) confirmed the representation of the actual Aβ peptides by HiLyte fluorophore. Ventricle injection performed at 3 dpf. Dotted white lines depict the border of optic tectum and hindbrain. (E-F) Fluorescence images of the whole-mount immunostained 3 dpf larvae with anti-pan-Aβ42 antibody (green) injected with mAβ42 (E) and oAβ42 (F), lateral view. White-dotted circles depict the pronephros regions. Neither HiLyte fluorophore nor 4G8-positive signals were barely detectable in the pronephros of the oAβ42 injected larvae, due to the insufficient peripheral clearance of oAβ42. Scale bars = 100μm. HB, hindbrain; hpi, hours post injection; OT, optic tectum.

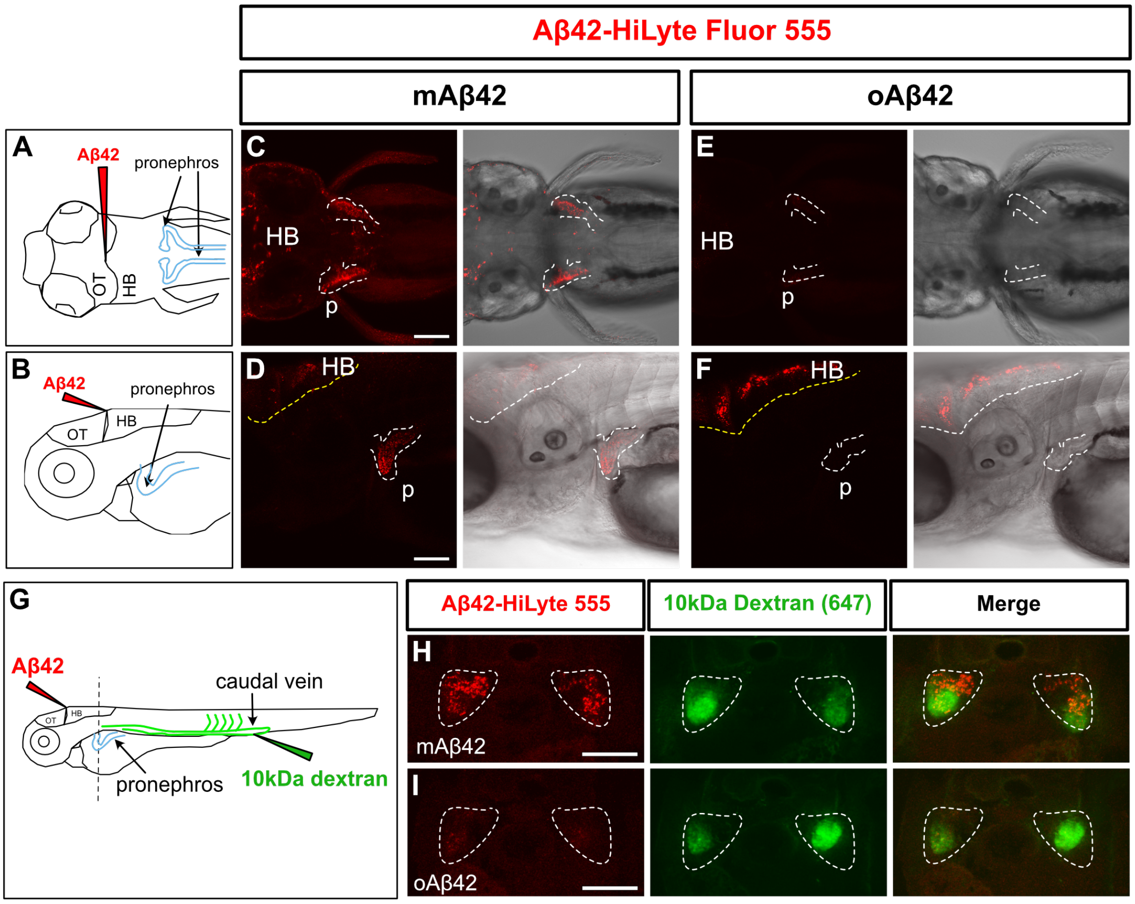

Figure S4. Cleared mAβ42 is transported to the peripheral pronephros. (A, B) A schematic diagram showing the brain and the pronephros of zebrafish larvae at 3 dpf with dorsal (A) and lateral (B) views. (C, D) Fluorescent (left) and bright field overlaying (right) images of mAβ42-injected 3 dpf larvae. Dorsal (C) and lateral (D) views. mAβ42 was seen in the pronephros region (white dotted lines in C and D). (E, F) Fluorescent (left) and bright field overlaying (right) images of oAβ42-injected larvae. oAβ42 was not detected in the pronephros region. (G) A schematic diagram of experimental setting for tracer injection. After mAβ42 or oAβ42 injected into ventricle, the dextran tracer was injected into the caudal vein. (H, I) Confocal images of transverse sections of the pronephric tubules of 3 dpf zebrafish larvae after injection of dextran with mAβ42 (H) or oAβ42 (I). Dotted lines indicate the outline of pronephric tubules. HB, hindbrain; OT, optic tectum; p, pronephros; Scale bars = 100 μm

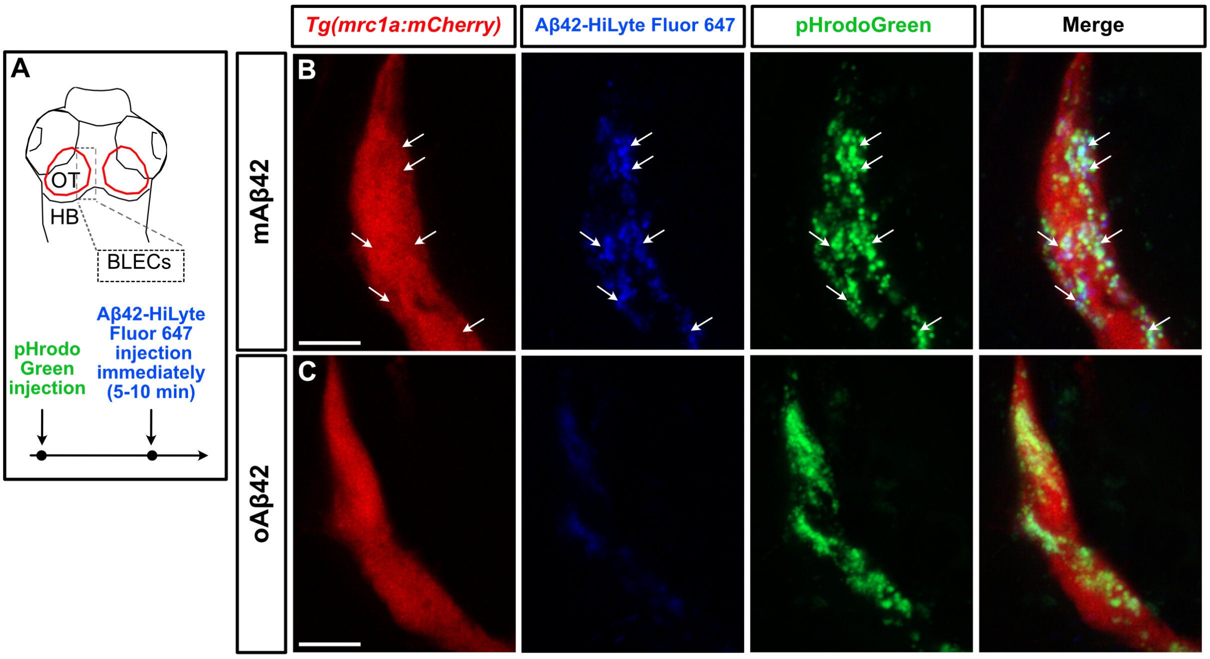

Figure S5. BLECs internalize mAβ42 with a pH sensitive dye pHrodoGreen. (A) A schematic diagram of zebrafish larval brain with dorsal view and experimental setting. Dotted box indicates the region of interest. Prior to mAβ42 or oAβ42 injection, pHrodoGreen was injected in the *Tg(mrc1a:mCherry)* transgenic line at 4 dpf. (B) Confocal images of *mrc1a*+ positive BLECs with mAβ42 (Hilyte Fluor 647) (B) or oAβ42 (C) and pHrodoGreen at 4 dpf. Scale bar =10 μm

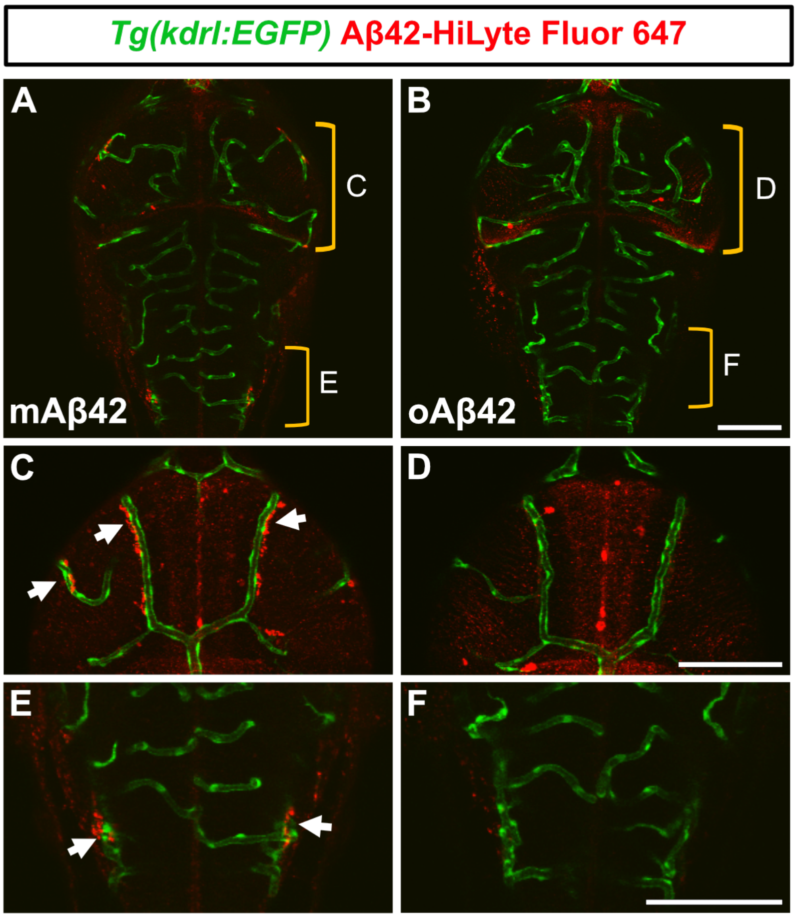

Figure S6. mAβ42, but not oAβ42, is localized adjacent to the cerebrovasculature. (A-F) Confocal fluorescence images of *Tg(kdrl:EGFP)* with mAβ42 (A, C, E) or oAβ42 (B, D, F) injection at 3 dpf. mAβ42 was detected proximal to the vasculature in the optic tectum (A, C) and the primordial hindbrain channels in the hindbrain (A, E), whereas oAβ42 was not (B, D, F). White arrows in C and E indicate Aβ42 fluorescence adjacent to *kdrl:EGFP+* endothelial cells. Scale bars = 100 μm.

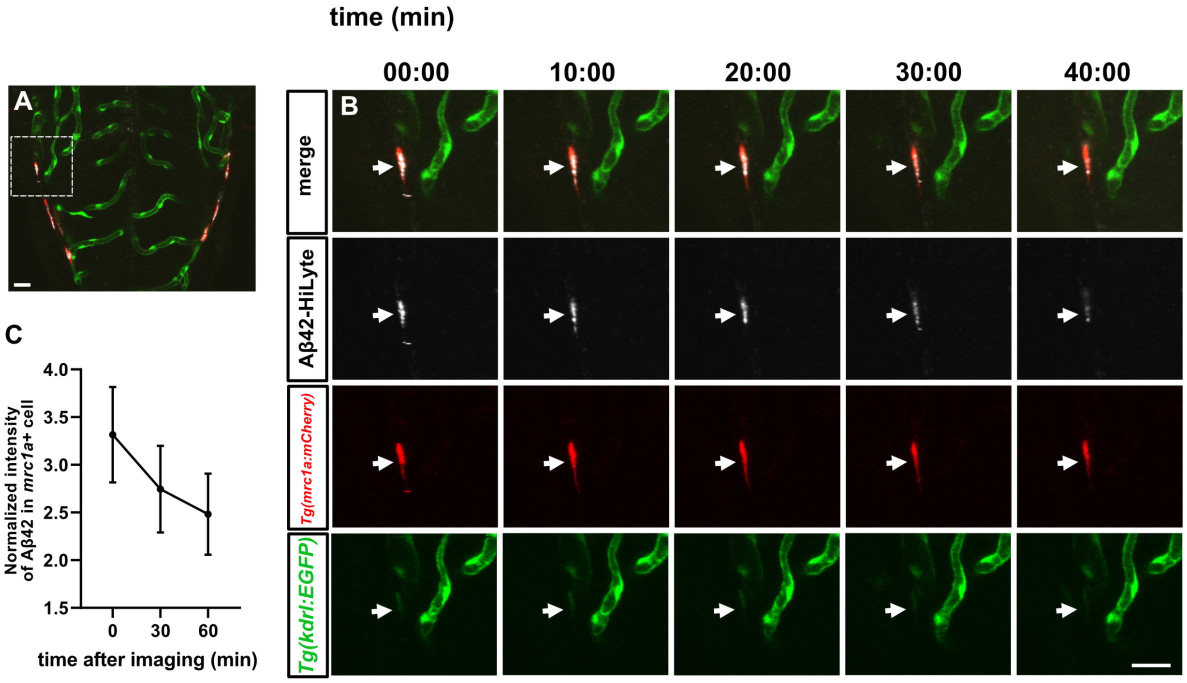

Figure S7. mAβ42 in the *mrc1a*+ positive cells gradually disappeared. (A) Still images of the time lapse video (Supple video) of double transgenic 3 dpf *Tg(mrc1a:mCherry); Tg(kdrl:EGFP)* injected with mAβ42 (HiLyte Fluor 647) at 4 hpi. (B) Time course montages of mAβ42 in the *mrc1a*+ BLECs for one hour. Arrows indicate mAβ42 with weakening intensity. (C) Time course quantification of mAβ42 intensity in the *mrc1a*+ BLECs normalized to non-fluorescents background. Similar findings were observed in four individual fishes. Data are presented as mean ± SEM. Scale bars = 20 μm.

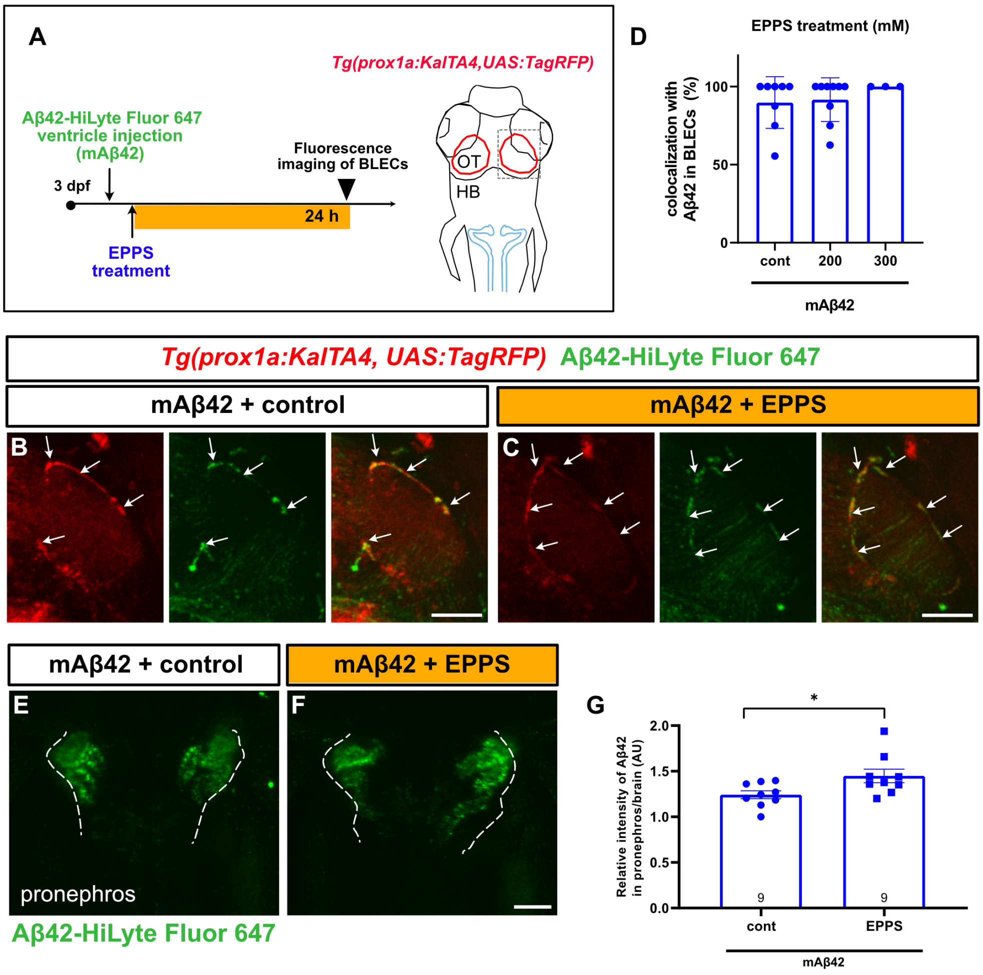

Figure S8. EPPS does not affect the co-localization of mAβ42 with BLECs. (A) A schematic diagram of EPPS treatment after mAβ42 (HiLyte Fluor 647) injection. (B, C) Confocal images of BLECs near the loop of the optic tectum in *Tg(prox1a:KalTA4, UAS:TagRFP)* after mAβ42 injection at 4 dpf. Vehicle-treated control (B) and EPPS (200 mM)–treated condition (C). White arrows indicate Aβ42 co-localized with BLECs. Scale bars = 50 μm. (D) Quantification of mAβ42 co-localization in BLECs upon EPPS treatment (%). (E, F) Confocal images of the pronephros after mAβ42 injection with EPPS treatment (F) or with vehicle treatment (E). (G) Quantification of the relative ratio of the intensity between the pronephros and brain after mAβ42 injection with or without EPPS treatment. Two tailed unpaired *t*-test, p= 0.0295. Data are presented as mean ± SEM. ns, not significant; *, p <0.05

Supplementary Video 1.

A time-lapse video of double transgenic line *Tg(kdrl:EGFP);Tg(mrc1a:mCherry)* larvae with m Aβ42 (HiLyte Fluor 647) at 3 dpf (10 min per frame, total 7 frames). (A) The hindbrain region of mAβ42 injected larvae. (B) An enlarged region in the hindbrain showed that the mAβ42 fluorescence in *mrc1a*+ adjacent to the vasculature gradually cleared. Scale bars = 20 μm. Data is representative of at least four independent experiments.
